# Calcium-Binding Generates the Semi-Clathrate Waters on a Type II Antifreeze Protein to Adsorb onto an Ice Crystal Surface

**DOI:** 10.3390/biom9050162

**Published:** 2019-04-27

**Authors:** Tatsuya Arai, Yoshiyuki Nishimiya, Yasushi Ohyama, Hidemasa Kondo, Sakae Tsuda

**Affiliations:** 1 Graduate School of Life Science, Hokkaido University, Sapporo 060-0810, Japan; tatarai0926@gmail.com; 2 Bioproduction Research Institute, National Institute of Advanced Industrial Science and Technology (AIST), Sapporo 062-8517, Japan; y.nishimiya@aist.go.jp (Y.N.); ohyama.y@aist.go.jp (Y.O.); 3 OPERANDO Open Innovation Laboratory, National Institute of Advanced Industrial Science and Technology (AIST), Tsukuba 305-8563, Japan

**Keywords:** antifreeze protein, ice-binding protein, crystal structure, Ca^2+^-binding, polypentagonal ice-like waters

## Abstract

Hydration is crucial for a function and a ligand recognition of a protein. The hydration shell constructed on an antifreeze protein (AFP) contains many organized waters, through which AFP is thought to bind to specific ice crystal planes. For a Ca^2+^-dependent species of AFP, however, it has not been clarified how 1 mol of Ca^2+^-binding is related with the hydration and the ice-binding ability. Here we determined the X-ray crystal structure of a Ca^2+^-dependent AFP (jsAFP) from Japanese smelt, *Hypomesus nipponensis*, in both Ca^2+^-bound and -free states. Their overall structures were closely similar (Root mean square deviation (RMSD) of Cα = 0.31 Å), while they exhibited a significant difference around their Ca^2+^-binding site. Firstly, the side-chains of four of the five Ca^2+^-binding residues (Q92, D94 E99, D113, and D114) were oriented to be suitable for ice binding only in the Ca^2+^-bound state. Second, a Ca^2+^-binding loop consisting of a segment D94–E99 becomes less flexible by the Ca^2+^-binding. Third, the Ca^2+^-binding induces a generation of ice-like clathrate waters around the Ca^2+^-binding site, which show a perfect position-match to the waters constructing the first prism plane of a single ice crystal. These results suggest that generation of ice-like clathrate waters induced by Ca^2+^-binding enables the ice-binding of this protein.

## 1. Introduction

Antifreeze proteins (AFPs) are structurally diverse macromolecules identified from variety of cold-adapted organisms such as fish, insects, plants, and microorganisms [1]. A common feature of AFPs is to construct an ice-binding site (IBS) on its surface, which possesses an ability to bind to specific ice crystal planes [2]. General ice is made of numerous single ice crystals that grow and tightly merge together. AFPs prevent such growing and merging as they coat the surface of each embryo crystal partly or entirely, leading the generation of a dispersion state of numerous small single ice crystals [3,4]. A micro-curvature created on each ice surface between the adsorbed AFP molecules decreases the non-colligative freezing point (*T*_f_), without a significant increase of the melting point (*T*_m_) [5]. The resultant temperature difference between *T*_f_ and *T*_m_ is termed thermal hysteresis (TH). With this activity, AFPs have been thought to avoid the cold damage of the host organisms [6].

A single ice crystal consists of hexagonally arranged water molecules that construct a three-dimensional lattice [7]. This lattice denoted I_h_ is characterized by three equivalent *a*-axes perpendicular to *c*-axis which create two basal- and six 1st prism planes, where the hexagonal arrangement is only observed in the basal plane. When I_h_ is cut along the *c*-axis so as to include two alternate corners, the created slice is called 2nd prism plane. The pyramidal planes are all the other slices of I_h_. Antifreeze proteins are capable of binding to these planes and modifies each crystal into a hexagonal plate, a hexagonal bipyramid, a hexagonal trapezohedron, a lemon-like morphology, or a sharpened needle [4,8,9]. Both TH and ice shaping are two representative abilities of AFP, and have a great potential for cryotechnology in industrial and medical fields [10,11].

The hydration waters organized into a polypentagonal but not a hexagonal arrangement were identified on the surface of a few AFP species [12,13]. These ice-like semi-clathrate waters were thought to merge with a 10–15 Å quasi-liquid layer [14] constructing an ice surface in a solution, in which water molecules are less well ordered compared with the hexagonally latticed waters [15,16]. Such an ice-binding manner with using the semi-clathrate ice-like waters was recently postulated for AFPs, and was called “anchored clathrate water (ACW) mechanism”. Garnham et al., 2011 initially proposed this mechanism based on the crystal structure determination for an AFP from Antarctic bacterium *Marinomonas primoryensis* (MpAFP) [17]. This bacterial AFP possesses linearly-arrayed hydration waters that exhibited space-match to latticed waters constructing the basal- and the 1st prism-planes of an ice crystal. Sun et al., 2014 and Mahatabuddin et al., 2018 further reported that the semi-clathrate waters on fish-derived AFPs construct polypentagonal water networks [12,13]. However, such polypentagonal waters were merely observed in the AFP structures, since their hydrophobic surfaces tend to be located in a face-to-face manner during the crystal-packing, leading to displacement of water molecules from their original positions [18]. It is therefore not clear whether the ACW mechanism is commonly adopted for all species of AFP.

Antifreeze proteins exhibit a remarkable structural diversity. The fish-derived AFPs can be categorized into five distinct groups (type I–IV AFPs, and antifreeze glycoprotein) based on their amino-acid sequence and structural property [1,2,3,19]. Type I AFPs is a 3 kDa alanine-rich α-helical protein, on which threonines are aligned with regular intervals on a protein surface. Type II AFP is a 14 kDa globular protein composed of both α-helices and β-strands with five disulfide-bonds. Type III AFP is a 7 kDa globular protein containing a compound IBS, in which two adjacent IBSs are inclined at 150° to each other. Type IV AFP is a four-helix bundle variant, though its expression was hardly detected in fish plasma [20]. Antifreeze glycoprotein (AFGP) consists of tripeptide repeats (Ala-Ala-Thr)_n_ (*n* = 4–50), in which the side-chain of threonine is glycosylated with β-d-galactosyl-(1,3)-α-*N*-acetyl-d-galactosamine. Among them, type II AFP is further sub-divided into two groups based on the requirement of 1 mol of Ca^2+^ for the TH and the ice-shaping activities. The Ca^2+^-dependent type II AFPs have been identified from Japanese smelt (*Hypomesus nipponensis*) (jsAFP) [21], rainbow smelt (*Osmerus mordax*) (smeltAFP), and Atlantic herring (*Clupea harengus*) (hAFP) [22]. In contrast, the Ca^2+^-independent AFPs have been identified from sea raven (*Hemitripterus americanus*) (srAFP) [23] and longsnout poacher (*Brachyopsis rostratus*) (BrAFP) [24]. Although the sequence identity of Ca^2+^-dependent and -independent species share only 40% of their sequences, the X-ray study showed that they are similar to each other [24], and are also similar to the Ca^2+^-dependent (C-type) lectin reference [25]. Although a location of IBS was not perfectly clarified for jsAFP, mutation studies originally suggested that it is located near the Ca^2+^-binding loop [26]. Further studies with docking simulations suggested that two Ca^2+^-coordinating residues (D94 and E99) as well as two polar residues (T96 and T98) with a protein-bound Ca^2+^ ion are involved in the ice-binding [27]. However, the manner of Ca^2+^-induced structural change and its relevance to the ACW mechanism are unknown. Here we determined X-ray crystal structures of Ca^2+^-dependent type II jsAFP in both Ca^2+^-bound and -free states, and discuss its ice binding mechanism through the observation of the ice-like hydration waters located on the former.

## 2. Materials and Methods

### 2.1. Preparation of Antifreeze Protein from Japanase Smelt and Thermal Hysteresis Measurements

Recombinant jsAFP was prepared as previously described [28]. Briefly, *Pichia pastoris* transformed with *pPICZα* (Thermo Fisher Scientific, MA, USA) containing His-tagged jsAFP gene was cultivated at 20 °C and separated from culture supernatant by centrifugation and filtration. The jsAFP in the culture supernatant was purified with Ni-NTA (QIAGEN, Hilden, Germany) column and following Superdex 200 column (GE- Healthcare, Amersham, UK). The TH activity was measured according to reference [29]. In short, a 1μl of AFP solution in glass capillary on Leica DMLB100 photomicroscope system (Leica Microsystems, Wetzlar, Germany) equipped with temperature controller was once frozen to form a multi-crystalline state of ice and gradually melt around 0 °C to form a single ice crystal. After obtaining this crystal, the temperature was decreased at a rate of 0.1 °C/min. The temperatures at which the ice-crystal started melting and growing were defined as the melting point (*T_m_*) and freezing point (*T_f_*), and TH was calculated as the difference between *T_m_* and *T_f_*.

### 2.2. Crystallization and Structural Analysis of Antifreeze Protein from Japanase Smelt

The crystallization of jsAFP was performed as described in reference [28]; it was generated with 0.1 M sodium acetate buffer (pH 4.0) containing 0.25 M ammonium sulfate and 8% PEG 3350 (Hampton research, CA, USA). It was found that the crystal belongs to the trigonal space group of *P*3_1_21 or its enantiomorph *P*3_2_21. The molecular replacement calculation revealed a clear location of one protein molecule in an asymmetric unit with space group *P*3_1_21. In the Ca^2+^-binding site, however, a poor electron density was observed for the possible position of Ca^2+^, which suggested a low occupancy of Ca^2+^ ion under the initial crystallization condition. This crystal was hence transferred into a pH-modified crystallization solution (pH 7.0) containing 2 mM of CaCl_2_, which successfully created the crystal of jsAFP that tightly binds 1 mol of Ca^2+^. To prepare the Ca^2+^-free jsAFP crystal, another crystal was soaked into the crystallization solution of pH 3.0 containing no CaCl_2_. Following the incubation for 24 h, the crystal was flash-cooled to 100 K. Diffraction data of Ca^2+^-bound and -free jsAFP were then collected using 1.0 Å radiation with the beamline NW12A at the Photon Factory, KEK, Tsukuba, Japan [30]. The collected diffraction data was processed using HKL-2000 [31] and CCP4 [32] software suites. The structure of jsAFP was determined by the molecular replacement method using MOLREP software [33], using the crystal structure of a Ca^2+^-independent type II AFP (BrAFP, PDB ID: 2ZIB) as a template. The calculated models of jsAFP were examined using Coot [34], and further refined using CNS [35] and REFMAC5 [36].

The protein structures in all figures were prepared using UCSF Chimera [37], in which hydrogen bonds were created with using the default setting. The docked model of jsAFP with the first prism plane of a single ice crystal was prepared manually according to the previous report [38] with slight modifications. The coordinate consisting of four layers of the first prism plane was prepared using VESTA [39]. One oxygen atom of the waters in the first prism plane was chosen as an origin of the rotation, and superimposed into one of the hydration waters on the IBS of Ca^2+^-bound jsAFP. The 1st prism plane was then rotated stepwise around the selected origin to achieve the maximal superimpositions of the waters located on the IBS and the ice 1st prism plane. The root mean square deviation (RMSD) value between these waters was used to determine the final structural model of the jsAFP-ice complex.

## 3. Results and Discussion

### 3.1. Antifreeze Protein from Japanase Smelt Inhibits Ice Crystal Growth when it Binds Ca^2+^

**Figure 1:**
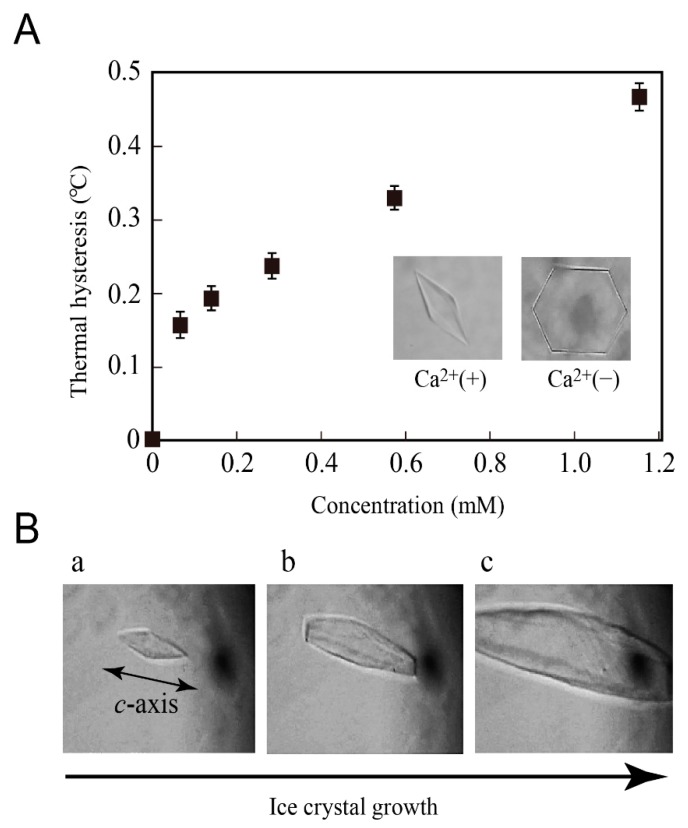
Thermal hysteresis (TH) and ice shaping activities of antifreeze protein from Japanese smelt (jsAFP). (**A**) Concentration-dependence of TH of jsAFP in the presence of 10 mM CaCl_2_. The measurement was triplicated to determine the averaged values (squares) with errors. The images show a single ice crystal presented in 1.0 mM jsAFP solution modified into a bipyramid with Ca^2+^ (left) and that modified imperfectly without Ca^2+^ (right). (**B**) Snapshots showing the process of “bursting” ice crystal growth observed for 0.1 mM of Ca^2+^-bound jsAFP. The jsAFP-binding to the 1st prism plane is supported by this crystal bursting along the *c*-axis direction.

Recombinant jsAFP was expressed with *P. pastoris* expression system and purified according to reference [28]. It was demonstrated that the N-linked glycosylation at 12th asparagine (N12) is not involved in the ice-binding [26]. Since such glycosylation generally prevents crystallization of a protein, we prepared its de-glycosylated mutant whose N12 is replaced with D, which is named jsAFP. In the presence of 10 mM of CaCl_2_, jsAFP modified a single ice crystal into a hexagonal bipyramid (Figure 1A), and lowered its ice-growth-initiation temperature that equals to *T*_f_. The *T*_m_ was separately evaluated at 0 °C. The TH value (TH = *T*_m_ − *T*_f_) vs. concentration shows the typical enzyme-kinetics-profile to reach 0.47 °C (Figure 1A). This value is identical to that obtained for a native sample [26].

Figure 1A is also similar to the TH profile obtained for another Ca^2+^-dependent type II AFP identified from herring (hAFP) [25], which shares approximately 80% sequence identity to jsAFP. When it decreased the temperature below *T*_f_, the ice crystal bipyramid (Figure 1B(a)) on which jsAFP molecules are accumulated showed a bursting ice crystal growth toward the *c*-axis direction (Figure 1B(b,c)). The Ca^2+^-free state of the jsAFP solution was prepared by addition of 20 mM EDTA. In this condition, jsAFP failed to form the hexagonal bipyramid even at higher concentrations such as 1.0 mM (14 mg/mL) (Figure 1A) [28]. No detectable TH activity was observed for this solution, indicating that jsAFP loses the ice-binding ability in the absence of Ca^2+^.

### 3.2. Antifreeze Protein from Japanase Smelt Targets First Prism Plane of a Single Ice Crystal

**Figure 2:**
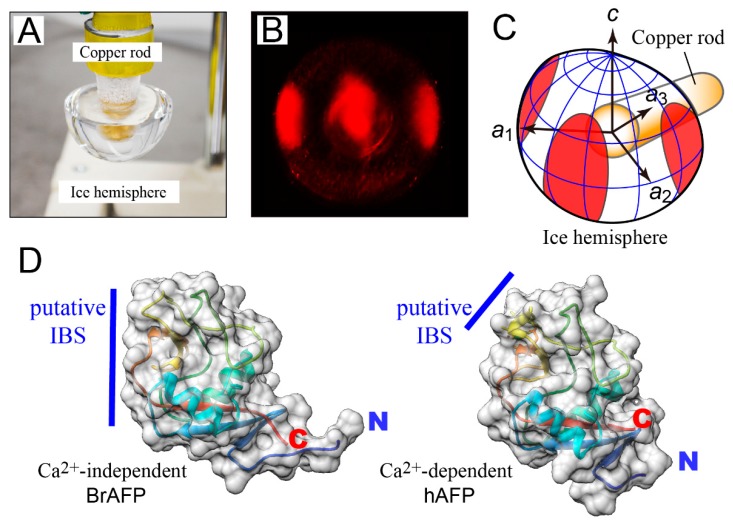
Analysis of fluorescence-based ice plane affinity (FIPA) and location of ice-binding site (IBS). (**A**) A single ice crystal hemisphere (ϕ = 2 cm) mounted on a frosty copper rod so as to face down its 1st prism plane. This hemisphere is soaked into a 0.1 mg/mL solution of florescence-labeled jsAFP. (**B**) The FIPA pattern observed on the ice crystal hemisphere under ultra violet (UV) light. (**C**) Illustration to show the location of the FIPA pattern on the ice hemisphere. The 1st prism plane is a region flanked by *a*_1_- and *a*_2_-axes, on which jsAFP molecules are adsorbed. (**D**) Comparison of putative IBSs of Ca^2+^-dependent antifreeze protein (AFP) from herring (hAFP) (PDB ID: 2PY2) and Ca^2+^-independent AFP from longsnout poacher *Brachyopsis rostratus* (BrAFP) (PDB ID: 2ZIB) viewed from almost the same direction, in which ribbon models are superimposed. Compared with the large putative IBS contributed by G57, I58, and a segment from P87 to H118 formed in BrAFP, a small IBS consisting of Q92, D94 E99, and D113 was assumed for hAFP.

The fluorescence-based ice plane affinity (FIPA) [40] was analyzed to clarify the target ice crystal plane of jsAFP. A single ice crystal hemisphere was attached onto a frosty copper rod so as to face down its 1st prism plane (Figure 2A). This ice hemisphere was then immersed into a 0.1 mg/mL solution of a fluorescence-tagged jsAFP containing 100 μM CaCl_2_, and held for 2 h to induce ice-binding of jsAFP. Following this immersion, three red-colored ellipses were observed on the ice hemisphere (Figure 2B), which indicates that jsAFP specifically binds to three 1st prism planes of a single ice crystal [4,40] as illustrated in Figure 2C. This is the first evidence demonstrating a target ice plane of the Ca^2+^-dependent type II AFP, and supports appropriateness of the previous ice-docking model of hAFP, for which 1st prism plane was assumed as the target plane [27]. In contrast, ice-binding to 2nd prism plane was suggested for a Ca^2+^-independent type II AFP from longsnout poacher *Brachyosis rostratus* (BrAFP) [24]. These data suggest that the ice-plane specificity is different between the Ca^2+^-dependent and -independent type II AFPs. This is in good agreement with the previous indications that their IBSs are formed differently; it consists of T96, T98, D94 and E99 for hAFP and of a segment P87–H118 plus G57–I58 for BrAFP (Figure 2D) [24,27].

### 3.3. Crystal Structure of Antifreeze Protein from Japanase Smelt with and without Ca^2+^

The X-ray structure of jsAFP in the Ca^2+^-bound state was determined with preparation of its crystal in a solution composed of 0.1 M sodium acetate buffer (pH 7.0), 0.2–0.25 M ammonium sulfate, 8% PEG 3350, and 2 mM CaCl_2_. For determination of the structure in the Ca^2+^-free state, the crystal was soaked into the above solution without CaCl_2_ for which pH was adjusted to 3.0, since 1 mol of Ca^2+^ is detached from jsAFP in the acidic condition. The jsAFP crystals were affected by neither pH nor Ca^2+^. The diffraction data was collected at 1.06 and 1.25 Å resolution for Ca^2+^-bound and -free states, respectively.

**Figure 3:**
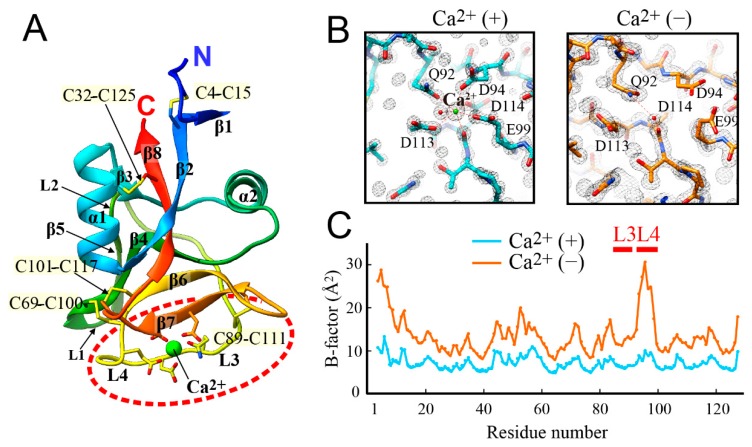
X-ray crystal structure of jsAFP. (**A**) The present determined structure of jsAFP in the Ca^2+^-bound state. The backbone conformation is represented by ribbon model with rainbow color. The model also includes the sticks showing five disulfide-bonds and 5 Ca^2+^-coordinating residues (Q92, D94 E99, D113, and D114). The Ca^2+^-binding site functioning as IBS is indicated by a red hatched circle. (**B**) Expanded views of the Ca^2+^-binding site of jsAFP with and without Ca^2+^. The 5 residues (sticks) are superimposed to the electron density map (black mesh). The Ca^2+^-bound form (left) only functions as IBS. (**C**) Comparison of B-factor (Å^2^) of the Cα-atom positions along the sequence of jsAFP between the Ca^2+^-bound (cyan) and -free (orange) states. The locations of the Ca^2+^-binding loops L3 (C89–Q92) and L4 (D94–E99) are indicated.

For jsAFP in the Ca^2+^-bound state, a structural model obtained with the molecular replacement method showed its clear density map including 1 mol of Ca^2+^. This model was further refined with including this Ca^2+^ ion, thereby elucidating the final structural model whose B-factor (Å^2^) is comparable between the backbone and side-chain atoms. This final model consists of 126 amino acid sequence from E3 to T128 as well as 289 water molecules with *R* factor of 0.154 and Free *R* factor [41] of 0.174 (PDB ID: 6JK4). Statistics for the data collection and refinement are summarized in Table 1. The overall structure of the Ca^2+^-bound jsAFP is composed of two alpha-helices (α1 and α2), eight beta-strands (β1–β8) and four loop regions (L1: C69–W75, L2: W77–T81, L3: C89–Q92, L4: D94–E99) (Figure 3A). The molecule is further stabilized by five disulfide-bonds (C4–C15, C32–C125, C69–C100, C89–C111 and C101–C117) and a hydrophobic core composed of aromatic and aliphatic residues. These features are mostly identical to that of the Ca^2+^-dependent hAFP (RMSD of Cα = 0.46Å) and of the Ca^2+^-independent BrAFP (RMSD of Cα = 0.71 Å) [24,27]. A Ca^2+^ ion was located at the molecular surface between β7 and a loop segment L3–L4 of jsAFP, and was coordinated with the side-chain oxygen atoms of Q92, D94 E99, D113, D114, a backbone oxygen atom of D114, and an oxygen atom of a water molecule.

The coordination geometry of Ca^2+^ in the jsAFP structure is highly similar to that in the hAFP structure. Among the six proline residues of jsAFP, P93 located between the two Ca^2+^-binding loops L3 and L4 adopts cis-conformation. This is conserved in all known three-dimensional structures of C-type lectins [25], so that it may play a crucial role in the construction of the Ca^2+^-binding site. The crystal structure of Ca^2+^-free jsAFP was determined to be 1.25 Å resolution (PDB ID: 6JK5), which consists of 125 amino acid sequence from C4 to T128 as well as 237 water molecules with an *R* factor of 0.18 and a free *R* factor [41] of 0.247. The crystal packing is identical to the Ca^2+^-bound state, which allowed an in-depth comparison between the two crystal structures. The similarity of their overall construction was indicated by a significantly low RMSD value (0.31 Å) between the two forms calculated using their 125 C_α_-atom positions. Structural changes were only observed near the Ca^2+^-binding site (Figure 3B). An electron density was observed at a distance of 1.6 Å from the Ca^2+^-position in the Ca^2+^-bound form (Figure 3B left). This density was assigned to a water molecule and refined with a reasonable B factor. This water forms hydrogen bonds to the oxygen atoms of the side-chain of Q92 as well as to the main chain of D114. The backbone conformation of the Ca^2+^-binding loop is also highly similar between the two jsAFP structures. In the Ca^2+^-free form, however, the side-chains of the loop excepting Q92 are moved away from the position of the vacant Ca^2+^ position (Figure 3B right). Side-chains of D94 and E99 are also not hydrogen-bonded to any water atom without Ca^2+^, while the connection to the side-chain OH-group of neighboring T96 located in L4. D113 is shifted approximately 1.3 Å apart from the Ca^2+^ position to form hydrogen bonds to the surface-bound waters on this molecule. The side-chain of D114 is roughly 140° rotated around its Cα-Cβ bond to approach the indole side-chain of W75. Due to a new hydrogen-bonding between D114 and W75, the indole ring adopts an opposite orientation compared with the Ca^2+^-bound structure, which is supported by our previous observation of Ca^2+^-induced change of the fluorescence intensity at 353 nm [26]. Figure 3C compares the B-factor (Å^2^) along the amino acid sequence between the Ca^2+^-bound and -free structures of jsAFP. A rapid molecular motion tends to raise the B factor of that segment, a significant increase evaluated for the Ca^2+^-binding loop L4 (D94–E99) suggests that this loop possesses a flexible nature only in the Ca^2+^-free form.

**Figure 4:**
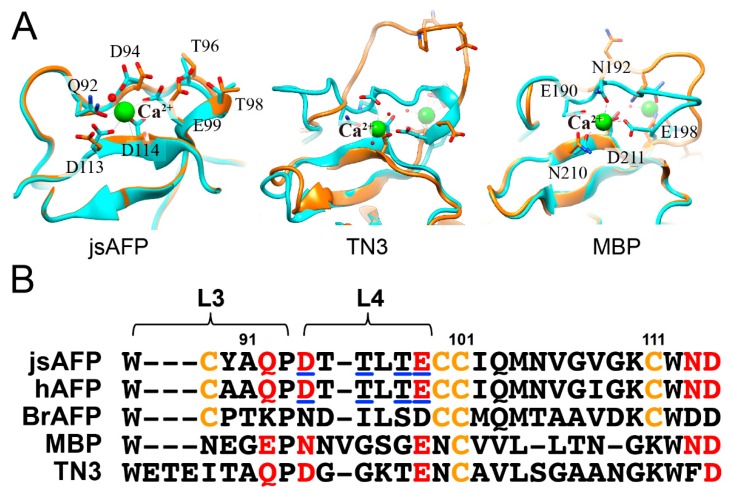
Structural comparison between jsAFP and the other C-type lectins. (**A**) The structures of a Ca^2+^-binding loop of jsAFP, tetranectin3 (TN3) and a mannose-binding protein (MBP). The backbone structure represented by ribbons colored cyan and orange for the Ca^2+^-bound and -free structures, respectively. The Ca^2+^-binding residues are shown in sticks, and Ca^2+^ ions are represented by green spheres. The backbone conformation of jsAFP is not changed by the releasing of Ca^2+^, while that of the others (TN3 and MBP) with 2 mol of Ca^2+^ is moved away from interior (cyan) to outside (orange). (**B**) Alignment of the amino acid sequence involved in the Ca^2+^-binding site (L3 and L4) of jsAFP, hAFP, BrAFP, MBP, and TN3 categorized into the C-type lectin. The residues known to participate in the disulfide-bonds and the Ca^2+^-binding site are colored in yellow and red, respectively. The putative ice-binding residues are underlined for AFPs.

Figure 4 compares the structure of Ca^2+^-binding loop (L3 and L4) of jsAFP with that of the two members of C-type lectin, tetranectin3 (TN3) and a mannose-binding protein (MBP) [43,44]. For jsAFP, conformation of the loop is almost identical between the Ca^2+^-free and -bound states (Figure 4A). In contrast, the loop of TN3 with 2 mol of Ca^2+^ is largely moved away from interior (cyan) to outside (orange) when Ca^2+^ ions are released. The Ca^2+^-binding loop of MBP associated with 2 mol of Ca^2+^ also becomes opened by the removal of Ca^2+^ (cyan→orange). In addition, *cis*-proline residue located at the junction between L3 and L4 of MBP, which corresponds to P93 of jsAFP, is isomerized to a trans-conformation by the Ca^2+^-removal. The loop region is probably pinned by the calcium ions in both TN3 and MBP, so that the region becomes free and moved toward outside when the ions are released. Significantly, the backbone conformation of the Ca^2+^-binding loop of jsAFP including cis-conformation of P93 is not virtually affected by removal of Ca^2+^. The reason for such structural holding might be due to construction of two disulfide-bonds, Cys69–Cys100 and Cys89–Cys111, which are highly conserved in type II AFPs but not in the other C-type lectins (Figure 4B). For example, a Ca^2+^-independent BrAFP also constructs the same loop conformation that involves cis-conformation of P93 in a similar way to jsAFP. Therefore, the whole backbone structure of jsAFP is not significantly affected by removal of 1 mol of Ca^2+^ ion. Then, why does jsAFP lose the ice-binding ability without Ca^2+^?

### 3.4. Ca^2+^-Bound Antifreeze Protein from Japanase Smelt Locates the Polypentagonal Ice-Like Waters

**Figure 5:**
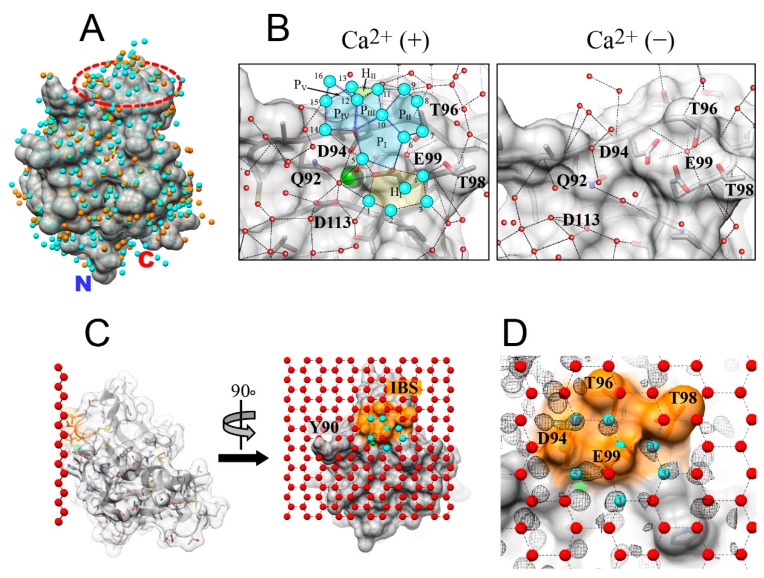
Ice-like semi clathrate waters located on the Ca^2+^-binding site. (**A**) Hydration waters located on the Ca^2+^-bound (cyan) and -free (orange) structures of jsAFP. The two structures are superimposed perfectly as their backbone conformation is highly similar to each other. An approximate position of the Ca^2+^-binding site functioning as IBS is indicated by a red hatched circle. (**B**) Polypentagonally arranged ice-like waters located on the Ca^2+^-binding site of jsAFP. The hydration waters are shown as red spheres, and those involved in the polypentagonal networks are represented by blue spheres. A total of five pentagonal waters (P_I_–P_V_) and one hexagonal waters (H_I_) are constructed on the Ca^2+^-bound structure of jsAFP, while not on the Ca^2+^-free structure. (**C**) Manual docking of the jsAFP structure to 1st prism plane of a single ice crystal. The oxygen atoms (red) of 1st prism plane perfectly fit to the Ca^2+^-binding site of jsAFP (left). The waters involved in the pentagonal network (blue) on the putative ice-binding residues (orange) share the positions of the oxygen atoms (red) constructing the ice plane (right). (**D**) An expanded view of the putative IBS docked with oxygen atoms constructing the 1st prism plane. The number labeled on the ice-like water (cyan) is the same as that labeled in (**B**). These waters (cyan) seem to be located in the clefts created between the ice-binding residues (D94, T96, T98, and E99).

According to the proposed ACW mechanism [13,17], AFP locates ice-like hydration waters on its molecule to easily merge with ice-water interface constructing the surface area of an ice crystal, and then freeze to a set of waters constructing the specific ice planes. Such ice-like waters were, however, not frequently observed since the hydrophobic IBS is merely exposed to the solvent [18,45]. In the present crystal structures, putative IBSs (Ca^2+^-binding loops) are exposed to the solvent in both Ca^2+^-bound and -free states. A total of 289 and 237 hydration waters were observed on the surface of the Ca^2+^-bound and -free structures of jsAFP, which are represented by spheres colored with cyan and orange, respectively (Figure 5A). The Ca^2+^-binding site, or the putative ice-binding site (IBS), is located in an area indicated with a hatched ellipse, on which it detected many hydration waters (cyan) only for the Ca^2+^-bound state. Significantly, a total of 16 waters are organized into an ice-like arrangement on the putative IBS (Figure 5B, left). They construct at least five pentagonal rings (P_I_~P_V_) and one hexagonal ring (H_I_) that are next to each other, in which a few oxygen atoms of the side-chain groups of jsAFP are also involved. Among them, the waters labeled 1, 4, 5, 6, 10, 12, and 14 directly anchor to the protein, while the others form second or third hydration layer on the protein. In contrast, no robust formation of such organized waters was detected in the Ca^2+^-free form (Figure 5B, right). It should be noted that the ice-like waters were not observed in the crystal structure of hAFP, which might be ascribed to a tight crystal packing around the Ca^2+^-binding loop. The ice-like water formations in the Ca^2+^-bound jsAFP structure seem to be stabilized by not only Ca^2+^-binding residues but also by the neighboring residues. For example, T96 and T98 are connected to E99 via the hydrogen-bonds to the waters 6 and 4. A previous mutation study demonstrated that substitution of T96 and T98 to hydrophobic residues such as alanine and valine decreased the TH activity, while substitution to serine did not [26]. The side-chain OH-groups of T96 and T98 are therefore thought to play a crucial role to anchor the water molecules via hydrogen-bonds, and function as a scaffold for the polypentagonal formation. Taken together, one may assume that the ice-binding of jsAFP occurs according to the ACW mechanism. This is the first observation of the ice-like water networks on the type II AFP molecule.

### 3.5. Ice-Like Waters can be Incorporated into the Quasi-Liquid Layer

Recently, an X-ray study showed that structure of a fish type III AFP hold pentagonally arranged ice-like waters in the vicinity of its IBS [13], and some of them exhibited position-match with those constructing the target ice-planes, such as the 1st prism and pyramidal planes of a single ice crystal. Since the 1st prism plane was suggested as a target plane of jsAFP (Figure 2), the Ca^2+^-bound structure of jsAFP was manually docked with 1st prism plane so as to superimpose the positions of the polygonal waters and those constructing the ice lattice as much as possible. The obtained docked model (Figure 5C) showed that waters 1, 3, 4, 5, 6, 10 (cyan) as well as the side-chain oxygen atoms of D94 and E99 perfectly match to the waters constructing the 1st prism plane, whose RMSD was less than 1.0 Å. In this model, methyl groups of A91, T96, and T98 and pyrrolidine group of P116 are oriented toward the hole in a hexagonal water ring, which was similarly observed in the ice-docking model of the type III AFP [13]. Such hydrophobic interactions between the IBS and ice structure presumably strengthen the van der Waals contacts between AFP and the ice crystal surface [46,47].

The residues of A91, T96, and T98 are conserved in the other type II AFPs, suggesting their importance for the ice-binding. It is also noted that Y90 causes steric hindrance with the ice-plane as shown in Figure 5C. Such a bulky residue might be unfavorable for ice-binding and is not conserved in the other type II AFPs. Hence, the side-chain orientation of this residue might be changed when jsAFP binds to ice crystal surface. We speculate that jsAFP first merges with quasi-liquid layer of ice crystal through the ice-like waters (Figure 5B), followed by a strengthening of the binding achieved by a slight re-orientation of the protein leading to the position-match to waters constructing the ice 1st prism plane. The other hydration waters located on the Ca^2+^-bound jsAFP might be also incorporated into the ice lattice concomitantly to its complexing with ice lattice, as suggested by molecular dynamics simulation [46,47]. The water-mediated ligand binding was also reported for the other C-type lectins [48,49]. Ewart et al. reported that substitution of Ca^2+^ ion with the other cations change both TH and ice-shaping activities of hAFP [50]. This may imply that the cation-substitution changes the ice-like water networks on the IBS of hAFP, but does not nullify the ice-like water formations.

### 3.6. Rigidity and Hydrophobicity May Relevant to the Ice-Like Water Formation

It was reported that structural rigidity and hydrophobicity of IBS are key determinants to forming the semi-clathrate waters [51]. The typical examples are insect AFPs constructing rigid β-helical structures stabilized by a hydrophobic core and many internal disulfide-bonds, on which the surface hydration waters are highly organized with regular intervals [52]. As shown in Figure 3C, the B-factors along the sequence of the Ca^2+^-bound jsAFP (cyan line) is lower than that of the Ca^2+^-free form (orange line), suggesting that 1 mol of Ca^2+^-binding rigidifies the overall structure of jsAFP. The hAFP in the Ca^2+^-free state was easily digested by trypsin while being less digested in the Ca^2+^-bound state [53], which also suggests a high flexibility to be exposed to the solvent in this type of AFP in the Ca^2+^-free state. For a Ca^2+^-binding loop (L4) from D94 to E99A of jsAFP, a significant difference of the B-factor was detected (Figure 3C), implying that its flexibility is lost by the binding of Ca^2+^. This loop region becomes more flattened, and a relevance between the flatness and IBS construction was also suggested [54]. The ice-like waters were only constructed on the IBS of Ca^2+^-bound jsAFP, suggesting that the flexible Ca^2+^-binding loop might be unfavorable for construction of the hydrogen bonding networks on this region. Indeed, the side-chains of D94, T96, T98, and E99 in the Ca^2+^-free jsAFP are not hydrogen-bonded to any water molecule.

Hydrophobicity of the molecular surface is also relevant to the protein hydration. Talon et al., 2014 reported that hydrophobic protein surface tends to be surrounded by the polygonal semi-clathrate waters, which avoids its exposure to the solvent [55]. They showed that substitution of such hydrophobic residues with acidic ones disrupts formation of the clathrate water networks. Indeed, the IBS of most the AFPs tends to be hydrophobic [2,45]. It is speculated that the Ca^2+^ ion in the jsAFP structure plays a role to neutralize the electric charge of the acidic residues of the IBS, which increases its hydrophobicity to lead and organize the polypentagonal ice-like waters. It is interesting that Ca^2+^-binding to jsAFP not only causes a slight conformational change of the side-chains near the IBS, but also fine-tunes the protein’s electricity and hydrophobicity to modify the hydration manner, leading to generating the ice-like waters to bind the host protein to an ice crystal surface.

## 4. Conclusions

In the present study, we determined the crystal structure of a Ca^2+^-dependent type II AFP from Japanese smelt denoted jsAFP, and made a structural comparison between its Ca^2+^-bound and -free forms to elucidate its ice-binding mechanism via Ca^2+^ ion. The obtained data revealed that binding of Ca^2+^ does not affect overall structural motif of jsAFP but alters its side-chain conformation as well as its rigidity of the Ca^2+^-binding loop, facilitating a formation of the polypentagonal ice-like waters on the IBS. These semi-clathrate waters exhibited perfect position-matches to the waters constructing the 1st prism plane, which was assigned to a target ice plane of jsAFP through the observation of the FIPA pattern on a single ice crystal hemisphere. This is the first report of Ca^2+^-induced ice-like water formations on the surface of AFP, which may suggest that AFPs inevitably use the ACW mechanism for its ice binding function.

## Figures and Tables

**Table 1:** Crystallographic data of X-ray structure of jsAFP.

Crystal	Ca^2+^-Bound Form (pH7)	Ca^2+^-Free Form (pH3)
Data Collection		
Space group	P3_1_21	
Unit-cell parameters (Å)	a = b = 65.95, c = 49.79	a = b = 66.03, c = 50.30
Beam line	Photon Factory AR-NW12A	
Wavelength (Å)	1.0	
Resolution range ^a^ (Å)	21.6–1.06 (1.12–1.06)	16.4–1.25 (1.32–1.25)
R_merge_^a, b^	0.045 (0.229)	0.052 (0.234)
Completeness ^a^ (%)	99.7 (100)	98.3 (100)
Multiplicity ^a^ (%)	10.5 (10.3)	10.1 (10.4)
<I/σ(I)> ^a^	10.1 (3.4)	8.5 (3.2)
Refinement Statistics		
Resolution range ^a^ (Å)	19.0–1.06 (1.087–1.06)	16.4–1.25 (1.282–1.25)
R factor ^a, c^	0.154 (0.213)	0.180 (0.247)
Free R factor ^a, c, d^	0.174 (0.227)	0.212 (282)
R.M.S bond length (Å)	0.017	0.016
R.M.S bond angles (°)	1.931	1.853
Residues	126	125
Number of non-hydrogen atoms		
Protein	993	975
Water	289	237
Other	1 (Ca^2+^)	5 (SO_4_^2−^)
Ramachandran plot ^e^ (%)		
Residues in favored regions	93	95
Residues in allowed regions	7	5
Residues in outliner regions	0	0
Average B factor (Å^2^)	11.0	17.0

^a^ Values in parentheses are for the highest resolution shell. ^b^ *R*_merge_ = $\sum \sum_{j}\left|\langle I\left(h\right)\rangle -I{\left(h\right)}_{j}\right|/\sum \sum_{j}\langle I\left(h\right)\rangle$, where 〈I(h)〉 is the mean intensity of a set of equivalent reflections. ^c^ *R* factor = $\sum \left|\left|F_{obs}\left(h\right)\right|-\left|F_{calc}\left(h\right)\right|\right|/\sum \left|F_{obs}\left(h\right)\right|$, where *F*_obs_ and *F*_calc_ are the observed and calculated structure factors, respectively. ^d^ 5% of the data was randomly chosen and used to calculate the free *R* factor. ^e^ Statistics were obtained from MolProbity [42].
